# 311. Heterologous immunity and host susceptibility to emerging alphaviral infections

**DOI:** 10.1093/ofid/ofad500.383

**Published:** 2023-11-27

**Authors:** Amy Y Vittor, Ruiyu Pu, Jean Paul Carrera, Maureen T Long, Sandra Lopez, Scott Weaver, John G Morris, David Pascual

**Affiliations:** University of Florida, Gainesville, Florida; University of Florida, Gainesville, Florida; Instituto Conmemorativo Gorgas de Estudios de la Salud, Panama, Panama, Panama; University of Florida, Gainesville, Florida; Instituto Conmemorativo Gorgas de Estudios de la Salud, Panama, Panama, Panama; University of Texas, Medical Branch, Galveston, Texas; University of Florida, Gainesville, Florida; University of Florida, Gainesville, Florida

## Abstract

**Background:**

In 2010, Madariaga virus (MADV) newly emerged in Darien, Panama, where Venezuelan equine encephalitis virus (VEEV) is endemic. We noted an inverse relationship between VEEV and MADV seroprevalence in humans, prompting the study of heterologous immunity in alphaviral infection.

**Methods:**

Using patient samples from Panama, we examined antibody-dependent cell mediated cytotoxicity (ADCC) and antibody-dependent cell mediated lysis assays (ADCL) to three viruses: Mayaro virus (MAYV), Madariaga virus (MADV), and the vaccine strain of Venezuelan equine encephalitis virus (TC83). We developed an ADCC assay using adherent target cells (human dermal fibroblasts), and high-throughput immunofluorescence microscopy. We determined ADCL activity using alphavirus-infected K562 cells and flow cytometry. We further defined the specificity of antibodies by plaque reduction neutralization tests, whole viral antigen and virus-specific recombinant glycoprotein (rGP) ELISAs.

**Results:**

Sera from alphavirus-exposed individuals demonstrated significantly greater ADCC and ADCL activity compared to alphavirus-naïve sera. Blocking CD16 abrogated this effect, confirming the role of natural killer cells in inducing ADCC and ADCL. The relationship is asymmetric; prior VEEV exposure yields a high degree of homologous and heterologous ADCC and ADCL activity, whereas MADV and MAYV exposure results in heterologous ADCC and ADCL activity no different than alphavirus-negative controls. We observed a strong association between ADCC/ADCL activity and whole viral antigen ELISA titers, but not rGP ELISA nor neutralization titers, suggests that the viral antigens that elicit ADCC and ADCL may be nonstructural proteins.

**Conclusion:**

Here we demonstrate that VEEV antibodies are active against heterologous alphaviral infections *in vitro* by ADCC and ADCL, but not by neutralization. This has implications for vaccine development. Eliciting an antibody response that is not only neutralizing, but also generates antibodies with ADCC and ADCL activity may produce broader protection against viral variants or antigenically similar viruses. Additionally, these results provide insight into immunological mechanisms underlying the emergence potential of a novel virus.

**Disclosures:**

**All Authors**: No reported disclosures

